# Investigating the impact of CBT on reducing impulsive clothing purchases among youths: A cognitive distortion perspective

**DOI:** 10.1097/MD.0000000000048798

**Published:** 2026-05-15

**Authors:** Ekwutosi Beatrice Oluah, Uzoamaka C. Ogor, Elizabeth Nkechi Ugwu, Margaret Ajanigo Atadoga, Daniel Okon, Damian C. Ncheke, Lilian C. Ozoemena, Ngozi Ngwanna Awoke, Uche Hope Ekwueme, Nkechi T. Egenti, D. C. Ezeuhara, Oluchi P. Ofozoba, Chinonye Lilian Opara, Daniel Omutal, Patience Nwobodo

**Affiliations:** a Department of Home Science & Management, University of Nigeria, Nsukka, Nigeria; b Department of Mass Communication, University of Nigeria, Nsukka, Nigeria; c Department of Home Economics, Federal College of Education, Eha-Amufu, Enugu, Nigeria; d Department of Food, Nutrition and Home, Prince Abubakar Audu University, Anyigba, Kogi, Nigeria; e University of Bamenda, Bambili, Northwest Region Cameroon, Cameroon; f Department of Counseling and Human Development Studies, University of Nigeria, Nsukka, Nigeria; g Department of Guidance and Counselling, Alex Ekwueme Federal University, Ndufu Alike, Ebonyi, Nigeria; h Department of Educational Foundations, University of Nigeria, Nsukka, Nigeria; i Department of Guidance and Counseling, Nnamdi Azikiwe University Awka, Nigeria; j Department of Education, Federal College of Education (Technical), Isu, Ebonyi, Nigeria.

**Keywords:** CBT, cognitive distortion, impulsive clothing purchases, Nigeria, youths

## Abstract

**Background:**

Impulsive buying behavior among youths, particularly in relation to clothing, represents a growing psychological and behavioral concern, often driven by cognitive distortions and difficulties in emotion regulation. Cognitive behavioral therapy (CBT) has shown promise in addressing maladaptive cognitions and behaviors; however, its targeted application to impulsive clothing purchases remains underexplored. Therefore, this study examined the effect of CBT on impulsive clothing buying behavior among young adults, with a specific focus on cognitive distortions and emotion regulation difficulties.

**Methods:**

A total of 122 eligible participants (aged 18–35 years) were recruited through purposive and convenience sampling from local communities, youth centers, and online platforms. Participants were randomly allocated via computer-generated randomization to either a CBT intervention group or a waitlist control group (1:1 ratio). Following attrition, 113 participants (53 in the CBT group and 60 in the control group) completed the study and were included in the final analysis. Only gender information was obtained at baseline, as participants were unwilling to provide additional demographic details. The intervention was delivered over 8 weekly sessions (90 minutes each) by licensed clinical psychologists trained in cognitive-behavioral approaches, under the supervision of a senior CBT consultant to ensure intervention fidelity. The CBT manual was adapted from A Therapist’s Guide to Brief Cognitive Behavioral Therapy. Standardized measures included the Clothing-Related Impulsive Buying Scale, the Clothing-Related Cognitive Distortions Scale, and the Clothing-Related Difficulties in Emotion Regulation Scale. Data were analyzed using analysis of covariance to assess group differences while controlling for pretest scores.

**Results:**

The CBT group demonstrated a statistically significant reduction in impulsive clothing buying behavior (*P* < .001), cognitive distortions (*P* < .01), and emotion dysregulation (*P* < .01) compared with the control group. Effect sizes were moderate to large, indicating a meaningful therapeutic impact.

**Conclusion:**

CBT delivered by trained and supervised clinical psychologists was effective in reducing impulsive clothing buying behavior by addressing cognitive distortions and enhancing emotion regulation. These findings highlight the potential of tailored CBT interventions for managing impulsive buying tendencies, particularly in fashion-related contexts.

## 1. Introduction

The phenomenon of impulsive purchasing, particularly in relation to apparel, has garnered significant scholarly interest due to its emotional, psychological, and financial ramifications. Characterized as an unplanned and spontaneous desire to make immediate purchases without comprehensive consideration, impulsive buying is predominantly influenced by emotional arousal rather than logical reasoning.^[1,2]^ While not inherently pathological, persistent and uncontrollable impulsive buying can result in considerable adverse effects, such as the accumulation of debt, emotional turmoil, and diminished well-being. Purchases of clothing are particularly susceptible to impulsivity due to their strong links to self-perception, identity development, and societal validation.^[3–5]^ Factors such as marketing strategies, social media influence, peer expectations, and internal issues like low self-esteem and emotional distress often catalyze impulsive buying behaviors related to clothing. Recent studies have increasingly highlighted the significance of difficulties in emotion regulation as a fundamental factor contributing to impulsive purchasing behaviors.^[6–8]^ Emotion regulation refers to the capacity to monitor, assess, and respond appropriately to emotional experiences. Individuals lacking effective emotion regulation skills may engage in impulsive buying as a means of coping with negative emotions such as boredom, sadness, or anxiety. However, this behavior often intensifies emotional distress over time, leading to a cyclical pattern of impulsivity and emotional dysregulation.^[8]^

The prevalence of impulsive and compulsive buying behavior varies across populations and contexts, as demonstrated by multiple studies. A study found an inverted U-shaped trend in compulsive buying among 2439 Chinese adults, with 11.3% prevalence in emerging adulthood (18–29 years), 18.5% in early adulthood (30–39 years), and 8.1% in middle adulthood (40–59 years).^[9]^ Similarly, another examined Indian consumers during the COVID-19 pandemic and reported moderate to high impulsive buying tendencies, with mean scores ranging from 2.62 to 3.54 on key indicators.^[10]^ Social media influence and pandemic-related stress explained 44.5% of the variance in impulsive buying. Complementing these findings, a US national survey estimated the prevalence of compulsive buying disorder at 5.8%, affecting both men (5.5%) and women (6.0%) nearly equally.^[11]^ In Europe, prevalence rates range from 3% among adults to as high as 8% among youth,^[12]^ while a French study found that 32.5% of customers at a Parisian department store exhibited signs of compulsive buying.^[13]^ Collectively, these findings underscore the global and context-dependent nature of impulsive and compulsive buying, highlighting the need for culturally sensitive interventions.

While earlier studies indicated distinct gender differences in impulsive buying behavior – where women were generally found to be more impulsive regarding clothing and accessories – recent research offers a more complex perspective. Current findings indicate that while women may exhibit greater impulsivity in fashion purchases, men also display similar impulsive tendencies in areas such as technology, leisure products, and online gaming.^[14]^ Social and cultural influences, particularly those concerning appearance and social standing, continue to affect gender-specific patterns of impulsive buying, although the disparity is diminishing as societal norms evolve.

Cultural and socioeconomic elements play a crucial role in shaping the frequency of impulsive buying behavior. Societies marked by consumerism, materialism, and significant advertising exposure, such as the United States, South Korea, and various European nations, exhibit elevated levels of impulsive purchasing.^[7,15]^ Conversely, there is a notable surge in impulsive buying within emerging markets, driven by increased access to smartphones, mobile commerce applications, and global marketing strategies.^[16–18]^ The convenience and immediacy of online shopping have further intensified impulsive clothing purchases, with tailored advertising and features like “one-click buying” diminishing self-regulation at the point of sale. In summary, impulsive buying, especially concerning clothing, is a widespread issue influenced by emotional vulnerabilities, cognitive biases, and sociocultural factors. The high incidence across various demographics underscores the urgent need for evidence-based psychological interventions, such as cognitive behavioral therapy (CBT), aimed at addressing the fundamental cognitive-emotional processes that underlie impulsive purchasing behaviors. Impulsive buying behavior, particularly in relation to clothing, has emerged as a significant psychological and social issue due to its widespread occurrence and adverse effects on individuals’ financial stability, emotional well-being, and overall life quality. Clothing, which is intricately linked to self-identity and social perception, is particularly susceptible to impulsive purchases driven by emotional rather than rational considerations.^[1,19]^ Purchases made during emotionally charged moments frequently lead to feelings of guilt, regret, and dissatisfaction, particularly when financial resources are limited or when the emotional needs that prompted the purchase remain unfulfilled. Over time, this cycle of emotion-driven impulsivity can worsen, resulting in chronic financial strain and psychological distress.^[2]^

Despite the growing body of evidence supporting the efficacy of CBT for behavioral addictions and compulsive shopping, there is a scarcity of research specifically targeting impulsive buying related to clothing.^[20]^ Clothing carries distinct emotional and symbolic significance tied to personal identity, appearance management, and perceived social belonging. Consequently, generalized interventions may not adequately address the intricate cognitive-emotional factors that drive impulsive clothing purchases.^[20–22]^ There is a pressing need for targeted interventions that specifically tackle the cognitive distortions and emotional vulnerabilities linked to clothing-related impulsivity.

CBT has been recognized as a potent psychological approach for addressing impulse-control challenges and behavioral addictions.^[5,19]^ CBT suggests that maladaptive cognitive patterns, including catastrophic thinking, emotional reasoning, and overgeneralization, perpetuate problematic behaviors. In the realm of impulsive purchasing, individuals frequently misinterpret internal signals (“I feel anxious; buying will alleviate this”) or external signals (“Everyone else appears better dressed; I must purchase this”) through distorted cognitive frameworks.^[23]^ By assisting individuals in recognizing, questioning, and altering these irrational thoughts, CBT promotes more intentional and rational decision-making processes, thereby mitigating impulsive behaviors.

This study aims to fill this gap by examining the effectiveness of a structured CBT intervention designed to mitigate impulsive clothing purchases. By focusing on both cognitive distortions and deficits in emotion regulation, the intervention aspires to provide a specialized treatment model that fosters emotional resilience and encourages more mindful consumer behavior. The outcomes of this research could inform the development of more tailored clinical and preventive strategies for managing impulsive buying tendencies, particularly in today’s consumer-oriented and image-centric society.^[19]^

CBT presents a valuable framework for addressing impulsive purchasing behaviors by focusing on the cognitive and emotional processes that underpin them. CBT is based on the understanding that maladaptive thought patterns can adversely affect emotional states and behaviors; therefore, altering these dysfunctional cognitive frameworks can facilitate improved emotional regulation and behavioral management.^[21,24,25]^ Individuals who frequently engage in impulsive clothing purchases often display cognitive distortions such as emotional reasoning (“If I feel good about it, it must be appropriate to buy it”), catastrophizing (“If I don’t purchase this immediately, I will miss out forever”), and overgeneralization (“Buying clothes consistently enhances my happiness”). CBT aims to deconstruct these distorted cognitive patterns and substitute them with more balanced, evidence-based reasoning, thereby fostering more intentional and thoughtful purchasing behaviors. Furthermore, difficulties in emotion regulation are increasingly acknowledged as significant factors contributing to impulsive buying tendencies. Inadequate emotional regulation – characterized by the inability to effectively monitor, assess, and adjust emotional responses – has been associated with a greater dependence on maladaptive coping mechanisms, including impulsive buying.^[4,8,26]^ For individuals experiencing negative emotional states such as anxiety, boredom, or sadness, engaging in clothing shopping may offer temporary emotional relief, albeit at the expense of reinforcing impulsivity and exacerbating emotional vulnerability. Through CBT interventions that emphasize emotion regulation training, individuals can acquire healthier strategies for managing their emotions, thereby diminishing their reliance on shopping as a means of emotional self-soothing.

While CBT has shown significant effectiveness in addressing behavioral addictions and broader compulsive buying behaviors, there is a scarcity of research specifically targeting impulsive clothing purchases. Clothing holds unique psychological implications tied to self-expression, social comparison, and identity management, indicating that interventions should focus on these particular emotional and cognitive triggers instead of treating impulsive buying as a uniform issue.^[19,27–29]^ Therefore, this study is justified by the necessity to create and assess a specialized CBT intervention aimed at mitigating impulsive clothing purchases by tackling both cognitive distortions and emotional regulation deficits. By concentrating on these interrelated psychological factors, this research aspires to enhance the understanding of the mechanisms that drive impulsive clothing buying and to aid in the formulation of more tailored and effective therapeutic approaches for managing this increasingly common behavior. The study specifically aims to evaluate the influence of CBT on impulsive clothing purchasing behavior among participants, analyze the effect of CBT on cognitive distortions associated with clothing purchases, and assess the impact of CBT on participants’ emotional regulation skills in relation to purchasing behavior.

### 1.1. Research questions

1. What is the effect of CBT on impulsive clothing purchases among participants?
2. How does CBT impact cognitive distortions associated with impulsive clothing buying?
3. What is the effect of CBT on emotional regulation in relation to impulsive buying behavior?

### 1.2. Research hypotheses

H_01_: There is no significant difference in impulsive clothing purchasing behavior between participants exposed to CBT and those not exposed.

H_02_: There is no significant effect of CBT on cognitive distortions related to impulsive clothing purchases.

H_03_: There is no significant difference in emotional regulation skills between the CBT group and the control group.

## 2. Research design

This study adopted a randomized controlled trial (RCT) design utilizing a pretest–posttest control group approach to examine the effect of CBT on impulsive clothing purchases, cognitive distortions, and emotion regulation among young adults. Participants who met the inclusion criteria were randomly assigned to either the CBT intervention group or the waitlist control group using a computer-generated randomization sequence. The RCT design provided a robust framework for evaluating changes attributable to the intervention while minimizing bias and controlling for potential confounding variables. The experimental group received an 8-week CBT program that focused on cognitive restructuring, impulse-control techniques, and emotional regulation strategies relevant to consumer decision-making. The waitlist control group received no intervention during the active phase but was offered the same CBT program after the completion of data collection. This approach ensured ethical fairness and maintained participant engagement. Data were collected at 2 time points: baseline (pretest) and immediately after the intervention (posttest).

### 2.1. Randomization and allocation concealment

The random allocation sequence was generated by an independent statistician who was not involved in participant recruitment or assessment. The sequence was created using a computerized random number generator in Microsoft Excel and implemented in a 1:1 allocation ratio (CBT group vs control group) without stratification. To maintain allocation concealment, the independent statistician prepared sequentially numbered, opaque, sealed envelopes containing group assignments. Each envelope corresponded to a unique participant identification number and was opened only after the participant had completed baseline assessments and provided informed consent. This procedure ensured that neither the participants nor the recruitment team could predict group assignments, thus minimizing selection bias.

### 2.2. Blinding

While participants were aware of their group allocation due to the nature of the behavioral intervention, outcome assessors and data analysts were blinded to group assignments throughout data collection and statistical analysis. Group codes (A/B) were used to anonymize datasets during analysis to preserve objectivity and reduce experimenter bias.

### 2.3. Ethical considerations

This study adhered to the ethical principles outlined in the Declaration of Helsinki and received formal approval from the Institutional Review Board of the Department of Counselling and Human Development Studies, University of Nigeria Nsukka (Ref: UNN/ED/CHDS/2024/022). Ethical clearance ensured that all procedures conformed to established standards for research involving human participants.

All participants were fully informed about the purpose, procedures, potential risks, and anticipated benefits of the study. Informed consent was obtained in writing before enrollment, and participants were reminded of their right to withdraw from the study at any stage without any form of penalty or loss of benefit. Confidentiality of participant information was maintained throughout the research process. Data were anonymized using identification codes, and all records were securely stored in password-protected digital files accessible only to authorized members of the research team.

### 2.4. Trial registration

The RCT was not registered prospectively prior to participant recruitment. This was due to administrative delays associated with obtaining institutional clearance and the limited availability of a national trial registry for behavioral interventions at the time of study initiation. However, full trial documentation, including the study protocol, intervention manual, randomization procedures, and statistical analysis plan, was prepared before data collection commenced and subsequently archived with the institutional ethics committee. Retrospective registration has been completed to ensure transparency and reproducibility.

## 3. Participants

A total of 200 young individuals were initially identified as potential participants, anticipating an attrition rate of approximately 20%. After eligibility screening, 122 participants met the inclusion criteria and were randomized equally into 2 groups of 61 participants each. Following attrition, 113 participants successfully completed the study and were included in the final analysis comprising 53 participants in the CBT group and 60 in the waitlist control group. The final sample size exceeded the minimum requirement established by an a priori power analysis conducted using G*Power version 3.1.9.7, which indicated that 51 participants per group (total N = 102) would be sufficient to detect a medium effect size (*d* = 0.5) at α = .05 and power = .80.

Participants were recruited through purposive and convenience sampling from local communities, youth centers, and online platforms such as social media networks and digital forums. Recruitment strategies included flyers, online advertisements, and referrals via peer networks.

### 3.1. Inclusion criteria

Eligible participants were required to:

1. Be between 18 and 35 years of age a demographic associated with heightened susceptibility to impulsive clothing purchases;
2. Score between 36 and 45 on the Clothing-Related Impulsive Buying Scale (CRIBS), indicating moderate to high impulsivity;
3. Score between 144 and 180 on the Clothing-Related Difficulties in Emotion Regulation Scale (CRDERS), suggesting significant emotional regulation challenges;
4. Demonstrate proficiency in English sufficient for comprehension of intervention materials; and
5. Provide informed consent and agree to complete all intervention and assessment sessions.

### 3.2. Exclusion criteria

Exclusion criteria included:

- Current participation in psychotherapy or counseling;
- Diagnosis of severe psychiatric or neurological disorders that could impair cognitive functioning; and
- Current use of psychoactive medication affecting impulse control or emotional regulation.

All participants underwent an initial screening involving a structured interview and background questionnaire to verify eligibility. Randomization was conducted immediately following the screening and baseline assessment. Throughout the study, 8 participants from the CBT group and one from the control group withdrew voluntarily, citing scheduling constraints. Participant confidentiality, anonymity, and voluntary participation were upheld throughout the study, and no adverse events were reported.

### 3.3. Measures

To assess the core variables of the study, impulsive buying behavior, cognitive distortions, and difficulties in emotion regulation standardized self-report instruments were used. These measures were selected based on their strong psychometric properties and relevance to the constructs being investigated.

#### 3.3.1. The Clothing Related Impulsive Buying Scale

The CRIBS was adapted from the original Impulsive Buying Scale (IBS) developed by Rook and Fisher.^[30]^ While the IBS was originally designed to measure general tendencies toward impulsive purchases, the CRIBS was specifically modified to focus on clothing-related purchasing behaviors. The CRIBS comprise 9 items, each rated on a 5-point Likert-type scale ranging from 1 (strongly disagree) to 5 (strongly agree). The scale captures participants’ tendencies to engage in unplanned and emotionally driven clothing purchases. Sample items include: “I often buy clothes spontaneously,” “Sometimes I purchase clothing based on how I feel at the moment,” and “I buy clothes on impulse without considering whether I need them.” This adaptation allows for more context-specific assessment, enhancing the scale’s relevance to the current study. The original IBS has been widely used in consumer behavior research and has demonstrated strong psychometric properties, including a Cronbach alpha of 0.88 and high construct validity. In this study, the CRIBS was subjected to expert review for content validity, ensuring its contextual alignment with clothing consumption behaviors.

#### 3.3.2. The Clothing Related Cognitive Distortions Scale (CRCDS)

To assess the role of dysfunctional thinking patterns in clothing-related impulsivity, the study employed the CRCDS. This instrument is a modified version of the Cognitive Distortions Scale (CDS) developed by Covin et al,^[31]^ which measures the frequency of distorted cognitive patterns that influence emotional and behavioral responses. The CRCDS consists of 20 items rated on a 7-point scale ranging from 1 (never) to 7 (all the time). It evaluates 2 primary domains: interpersonal distortions and achievement-related distortions. Specific cognitive distortions assessed include catastrophizing, mind reading, personalization, overgeneralization, and dichotomous thinking. Example items include: “If I fail partly, it is as bad as being a complete failure” and “If someone disagrees with me, it means they don’t like me.” These thought patterns are conceptually linked to irrational beliefs that can fuel impulsive purchases, particularly when individuals seek validation or relief through clothing acquisition. The CDS has demonstrated excellent internal consistency, with Cronbach alpha values of 0.93 in nonclinical samples and 0.92 in clinical populations. It also shows strong correlations with established measures of depression and anxiety, reinforcing its validity. In this study, items were reviewed and refined to emphasize distortions related to appearance, self-image, and fashion-based decision-making, thus enhancing the instrument’s sensitivity to the study context.

To establish psychometric reliability within the current sample, the adapted CRCDS was subjected to internal consistency analysis, yielding a Cronbach alpha coefficient of 0.89, indicating high reliability. The scoring procedure remained consistent with the original CDS, with higher total scores reflecting greater endorsement of clothing-related cognitive distortions. This adaptation and validation process ensured that the CRCDS was both culturally relevant and psychometrically sound for assessing cognitive distortions linked to impulsive clothing acquisition among the study population.

#### 3.3.3. The Clothing Related Difficulties in Emotion Regulation Scale (CRDERS)

The third instrument used in the study is the CRDERS, adapted from the Difficulties in Emotion Regulation Scale by Gratz and Roemer.^[32]^ Emotional dysregulation has been identified as a significant contributor to impulsive behaviors, particularly in consumer contexts where purchases may serve as a coping mechanism. The CRDERS contains 36 items, assessing 6 subdomains: nonacceptance of emotional responses, difficulties engaging in goal-directed behavior, impulse control difficulties, lack of emotional awareness, limited access to effective regulation strategies, and lack of emotional clarity. Respondents rate each item on a 5-point scale ranging from 1 (almost never) to 5 (almost always). Sample statements include: “When I’m upset, I feel out of control,” “I have difficulty making sense of my feelings,” and “When I’m upset, I believe I’ll remain that way for a long time.” The Difficulties in Emotion Regulation Scale has consistently demonstrated excellent psychometric properties, with a total score Cronbach alpha of 0.93 and subscale reliabilities ranging from 0.80 to 0.89. For the purposes of this study, the scale was tailored to reflect emotion regulation difficulties specifically related to clothing decisions and fashion-related stressors. This adaptation enhances its capacity to detect emotional patterns that may precipitate impulsive buying episodes. The adapted version was then pilot-tested on a small sample of adolescents to ensure comprehension and reliability within the study population. Internal consistency reliability (Cronbach alpha) for the adapted CRDERS in the present sample was 0.89, indicating strong reliability within the cultural context.

Collectively, these 3 instruments (CRIBS, CRCDS, and CRDERS) offer a robust, multidimensional assessment framework for understanding the cognitive and emotional drivers of impulsive clothing purchases among adolescents. Their targeted focus allows for a precise evaluation of the CBT intervention’s effectiveness in modifying maladaptive beliefs and emotional patterns associated with impulsive consumer behavior. Their established reliability and validity, along with the study-specific adaptations, support their appropriateness for use in this empirical investigation.

### 3.4. Intervention

The intervention employed in this study was adapted from A Therapist’s Guide to Brief Cognitive Behavioral Therapy developed by Cully and Teten.^[33]^ This manual provided a structured, evidence-based framework appropriate for addressing maladaptive behaviors, including impulsive clothing purchasing. The CBT program was specifically tailored to target the cognitive distortions and emotional dysregulation identified as core contributors to impulsive buying behavior among young adults.

The intervention comprised 8 structured group sessions, each lasting approximately 90 minutes and delivered once a week over an 8-week period. The sessions were facilitated by 2 licensed counseling psychologists, each holding doctoral qualifications in counseling and clinical psychology, with over 7 years of experience in administering evidence-based cognitive-behavioral interventions to young populations. Both facilitators possessed advanced professional certification in CBT and underwent additional orientation on consumer impulsivity and emotion regulation in preparation for this study.

To ensure treatment fidelity and professional oversight, the therapists were supervised weekly by a senior CBT practitioner and academic with more than 10 years of supervisory experience. Supervision sessions involved reviewing audio recordings of selected group sessions, discussing clinical challenges, and verifying adherence to the intervention protocol. A structured fidelity checklist was used after each session to ensure consistency in content delivery and maintenance of therapeutic integrity across groups.

The intervention model was divided into 3 integrated phases, psychoeducation, cognitive restructuring, and behavioral and emotional skills training, each designed to address a distinct aspect of the study variables.

#### 3.4.1. Psychoeducation phase (sessions 1–2): building awareness of impulsive buying and emotional dysregulation

This phase introduced participants to the CBT framework, with emphasis on the relationship between thoughts, emotions, and behaviors in the context of impulsive clothing purchases. Participants explored how cognitive distortions such as emotional reasoning (“I feel anxious, so buying clothes will help”), catastrophizing (“If I don’t buy this now, I’ll never get another chance”), and personalization influenced their shopping choices. Foundational concepts of emotional regulation, such as recognizing emotional triggers and understanding the role of impulsivity in decision-making, were also introduced.

#### 3.4.2. Cognitive restructuring phase (sessions 3–5): challenging and modifying distorted thinking patterns

During this phase, participants were guided through structured CBT activities to identify, challenge, and replace irrational beliefs concerning self-image, clothing preferences, and consumer behavior. Techniques such as thought records, Socratic questioning, and cognitive reframing were employed to dismantle negative automatic thoughts that perpetuated impulsive buying. This component directly addressed the cognitive distortion variable by promoting adaptive and realistic thinking patterns.

#### 3.4.3. Behavioral and emotional skills training phase (sessions 6–8): enhancing emotion regulation and impulse control

The final phase focused on equipping participants with practical tools for managing emotions and resisting impulsive urges. Strategies included urge surfing, delayed gratification exercises, and controlled shopping exposures, allowing participants to practice restraint in real or simulated shopping contexts. Emotion regulation skills, such as mindfulness-based coping, emotional labeling, and distress tolerance, were integrated to strengthen participants’ ability to modulate affective states that often trigger impulsive spending. Homework assignments, including emotion-monitoring diaries, shopping planning worksheets, and self-monitoring logs, reinforced skill application outside the therapy sessions.

Throughout the program, individualized CBT worksheets, feedback discussions, and group reflections were used to consolidate learning and encourage the transfer of skills to daily life. Intervention fidelity was further maintained through adherence monitoring and ongoing supervision. Participants in the control (waitlist) group did not receive any intervention during the study period but were offered the same CBT program upon completion of the post-intervention assessments to ensure ethical parity.

### 3.5. Therapist training and fidelity monitoring

All therapists involved in the intervention were doctoral-level counseling psychologists with advanced professional certification in CBT. Prior to the commencement of the study, the therapists underwent a 2-day refresher workshop conducted by a senior CBT consultant and academic expert. This workshop focused on the specific adaptation of the CBT framework to impulsive buying behavior, ethical considerations in group-based interventions, and procedures for maintaining treatment integrity.

To ensure consistent delivery and therapeutic quality, weekly clinical supervision was conducted by a consultant CBT practitioner with over 10 years of supervisory experience. During supervision, therapists reviewed session recordings and completed structured fidelity checklists to verify adherence to session protocols. Discussions centered on maintaining theoretical fidelity, handling group dynamics, and managing participant engagement issues. An independent observer, trained in CBT fidelity assessment, randomly reviewed 25% of recorded sessions to assess consistency with the treatment manual and rated adherence using a standardized checklist. Fidelity ratings consistently met or exceeded the preestablished criterion of 90% adherence, indicating strong reliability in intervention delivery. Regular supervision and fidelity checks collectively ensured both the internal validity of the study and the credibility of the intervention outcomes.

## 4. Procedure

After obtaining ethical approval from the Institutional Review Board, participants were recruited using purposive and convenience sampling methods through advertisements in local communities, youth centers, and social media platforms. Interested individuals completed an online screening that included demographic questions and the CRIBS to assess their tendency toward impulsive clothing purchases. Individuals who scored 40 or above on the CRIBS and met the inclusion criteria provided written informed consent to participate.

Eligible participants were subsequently randomized into either the CBT intervention group or the waitlist control group, utilizing a computer-generated simple randomization sequence with an equal allocation ratio of 1:1. The randomization sequence was produced by an independent research assistant who did not participate in the enrollment of participants or the collection of data. To maintain allocation concealment, sequentially numbered, opaque, sealed envelopes were employed, which had been prepared in advance by the same independent assistant. These envelopes were only opened after participants had completed their baseline assessments, ensuring that allocation blinding was preserved at the time of enrollment. Participants assigned to the intervention group engaged in an 8-week CBT program, which was adapted from A Therapist’s Guide to Brief Cognitive Behavioral Therapy^[33]^. The sessions were held weekly for 90 minutes each in small groups, led by trained clinical psychologists. The CBT program focused on identifying and restructuring cognitive distortions associated with impulsive clothing purchases and enhancing emotion regulation skills to mitigate impulsive buying behaviors.

Baseline assessments were conducted prior to the intervention, using the CRIBS, CRCDS, and CRDERS. Post-intervention assessments were administered immediately after the 8-week program for both groups using the same measures. All assessments were conducted face-to-face under the supervision of research assistants to ensure standardized administration and minimize missing data.

Attendance was recorded at each session, and participants experiencing any adverse emotional reactions were provided with supportive counseling or referral when necessary. After completing the study, the waitlist control group was offered the CBT intervention. All participant information was anonymized and securely stored. Data analysis was conducted to evaluate the effect of CBT on impulsive clothing purchases, cognitive distortions, and emotion regulation abilities.

## 5. Method of data analysis

Data were analyzed using IBM SPSS Statistics version 27. Descriptive statistics, including means, standard deviations (SDs), and frequencies, were calculated to summarize participants’ demographic characteristics and baseline scores. To ensure that there were no significant baseline differences between the groups, an independent samples *t*-test was initially conducted on pre-intervention scores for impulsive clothing purchases, cognitive distortions, and emotion regulation. Next, a series of one-way analysis of covariance (ANCOVA) was performed to evaluate the effect of CBT on impulsive clothing purchasing behavior, cognitive distortions, and emotion regulation post-intervention. ANCOVA was chosen to control for any potential differences in baseline scores between the intervention and control groups, ensuring that observed effects were due to the intervention rather than preexisting group differences. Effect sizes (partial eta squared) were calculated to determine the magnitude of group differences. Statistical significance was set at *P* < .05. Analyses were conducted on a complete-case basis due to 9 participants missing posttest data (7.4% attrition). Intention-to-treat sensitivity analyses produced comparable results.

## 6. Results

Table 1 presents the descriptive statistics of posttest scores on the CRIBS for participants in both the experimental and control groups, disaggregated by gender. The mean scores show that participants exposed to CBT recorded considerably lower impulsive buying tendencies (Male = 2.50; Female = 2.48) than those in the control group (Male = 4.27; Female = 4.35). Since higher scores indicate greater impulsivity in clothing purchases, these results suggest that the CBT intervention was effective in reducing impulsive buying behavior among participants. The smaller SDs and coefficients of variation in the experimental group indicate that the participants who received CBT responded to the intervention more consistently than those in the control group. Gender differences within each group were minimal, suggesting that the effect of CBT was similar for both male and female participants.

**Table 1:** Descriptive – Clothing Related Impulsive Buying Scale (CRIBS) POSTTEST.

Group	Gender	N	Mean	SD	SE	Coefficient of variation
Experimental group	Male	30	2.500	0.682	0.125	0.273
	Female	23	2.478	0.511	0.106	0.206
Control group	Male	37	4.270	1.097	0.180	0.257
	Female	23	4.348	0.982	0.205	0.226

There is no significant difference in impulsive clothing purchasing behavior between participants exposed to CBT and those not exposed.

To determine whether the observed difference between groups was statistically significant, an ANCOVA was conducted while controlling for pretest scores. The results in Table 2 reveal a statistically significant main effect of group, *F* (1108) = 113.60, *P* < .001, with a partial eta squared value of 0.513. This indicates that approximately 51% of the variance in posttest impulsive buying scores was attributable to the CBT intervention, confirming its substantial impact. Neither gender (*F* (1108) = 0.03, *P* = .864) nor the interaction between group and gender (*F* (1108) = 0.07, *P* = .792) was significant, showing that the CBT intervention was equally effective for both males and females. Furthermore, pretest scores did not significantly predict posttest results, suggesting that the observed improvements were primarily due to the CBT intervention rather than participants’ initial levels of impulsivity.

**Table 2:** ANCOVA – CRIBSPOSTTEST.

Cases	Sum of Squares	df	Mean square	*F*	*P*	η^2^_p_
Gender	0.023	1	0.023	0.030	.864	2.743 × 10^−4^
Group × gender	0.054	1	0.054	0.070	.792	6.466 × 10^−4^
CRIBSPRETEST	0.298	1	0.298	0.386	.536	0.004
Group	87.781	1	87.781	113.598	<.001	0.513
Residuals	83.455	108	0.773			

Type III sum of squares.

Overall, these findings demonstrate that CBT had a strong and positive effect on reducing impulsive clothing purchases among participants. The intervention appeared to enhance self-regulation and cognitive control over impulsive buying tendencies by helping participants identify and restructure irrational beliefs, modify automatic thoughts, and adopt more reflective purchasing decisions. This outcome supports the cognitive-behavioral theory that impulsive buying can be effectively managed by targeting the maladaptive cognitions and emotional triggers underlying the behavior.

Table 3 presents the descriptive statistics for posttest scores on the CRCDS among participants in the experimental and control groups. The mean scores reveal a marked difference between the groups. Participants exposed to CBT recorded substantially lower mean scores on cognitive distortions related to impulsive clothing purchases (Male = 1.93; Female = 1.91) compared with their counterparts in the control group (Male = 6.32; Female = 6.83). Since higher scores on the CRCDS reflect greater levels of cognitive distortions – such as irrational beliefs, faulty assumptions, and distorted thinking patterns associated with impulsive buying, the descriptive results suggest that the CBT intervention significantly reduced these maladaptive cognitions. The SDs and coefficients of variation are notably smaller in the experimental group than in the control group, indicating that participants who received CBT not only exhibited fewer cognitive distortions but also responded more uniformly to the intervention. Gender differences within the groups were minimal, suggesting that the beneficial effects of CBT on cognitive distortions were similar for both male and female participants.

**Table 3:** Descriptive – Clothing Related Cognitive Distortions Scale (CRCDS) POSTTEST.

Group	Gender	N	Mean	SD	SE	Coefficient of variation
Experimental group	Male	30	1.933	0.254	0.046	0.131
	Female	23	1.913	0.288	0.060	0.151
Control group	Male	37	6.324	1.454	0.239	0.230
	Female	23	6.826	0.491	0.102	0.072

The ANCOVA results presented in Table 4 further clarify the nature of this relationship. Controlling for pretest scores, the analysis revealed a statistically significant main effect of group, *F*(1108) = 745.57, *P*<.001, with a very large partial eta squared value (*η*^2^_*p*_ = 0.873). This indicates that approximately 87% of the variance in posttest cognitive distortion scores can be attributed to the CBT intervention, confirming a strong and meaningful impact. Neither gender (*F* (1108) = 1.99, *P* = .161) nor the interaction between group and gender (*F* (1108) = 2.30, *P* = .133) reached statistical significance, suggesting that the reduction in cognitive distortions was consistent across genders. The pretest covariate was also nonsignificant (*F* (1108) = 0.06, *P* = .800), indicating that pre-intervention differences did not account for the observed effects.

**Table 4:** ANCOVA – Clothing Related Cognitive Distortions Scale (CRCDS) POSTTEST.

Cases	Sum of Squares	Df	Mean square	*F*	*P*	η^2^_p_
Group	587.167	1	587.167	745.571	<.001	0.873
Gender	1.571	1	1.571	1.995	.161	0.018
CRCDSPRETEST	0.051	1	0.051	0.064	.800	5.968 × 10^−4^
Group × gender	1.808	1	1.808	2.296	.133	0.021
Residuals	85.054	108	0.788			

Type III sum of squares.

Overall, these findings demonstrate that CBT exerted a substantial and positive influence on the cognitive processes associated with impulsive clothing purchases. The intervention appeared to help participants identify, challenge, and modify irrational beliefs and cognitive distortions that drive impulsive spending behavior. This outcome aligns with the central tenets of cognitive-behavioral theory, which posit that maladaptive buying behaviors stem from distorted thinking patterns such as overgeneralization (“I must buy this to feel good”), personalization (“Others will value me more if I dress better”), or emotional reasoning (“I feel low, so shopping will make me happy”). By restructuring these faulty cognitions and enhancing cognitive control, CBT enabled participants to develop more rational and self-regulated attitudes toward purchasing decisions. Thus, the findings affirm that CBT can be a powerful tool in addressing the cognitive underpinnings of impulsive buying behavior.

What is the effect of CBT on emotional regulation in relation to impulsive buying behavior?

From Table 5, the descriptive statistics reveal a clear difference between the experimental and control groups. The experimental group (both male and female) recorded a constant mean score of 2.000 with no variability (SD = 0.000), indicating that participants in the CBT group exhibited strong and stable emotional regulation following the intervention. In contrast, the control group showed substantially higher mean scores, 4.162 (SD = 0.646) for males and 3.565 (SD = 0.843) for females, reflecting greater difficulties in emotion regulation after the posttest period. These differences suggest that CBT had a strong stabilizing effect on participants’ ability to manage emotions related to impulsive buying.

**Table 5:** Descriptive – Clothing Related Difficulties in Emotion Regulation Scale (CRDERS) POSTTEST.

Group	gender	N	Mean	SD	SE	Coefficient of variation
Experimental group	Male	30	2.000	0.000	0.000	0.000
	Female	23	2.000	0.000	0.000	0.000
Control group	Male	37	4.162	0.646	0.106	0.155
	Female	23	3.565	0.843	0.176	0.237

Table 6 presents the ANCOVA results, which statistically confirm this pattern. The main effect of group was highly significant, *F*(1108) = 335.157, *P* < .001, with a very large effect size (*η*^2^_*p*_ = 0.756). This indicates that CBT had a substantial and meaningful effect on improving emotional regulation among participants, even after controlling for pretest scores. The gender effect was also significant, *F*(1108) = 8.728, *P* = .004, *η*^2^_*p*_ = 0.075, suggesting that males and females differed somewhat in their emotional regulation outcomes, though the effect was moderate. Moreover, the interaction between group and gender was significant, *F*(1108) = 8.367, *P* = .005, *η*^2^_*p*_ = 0.072, implying that CBT’s impact varied slightly across genders – perhaps indicating that one gender benefitted more strongly from the intervention.

**Table 6:** ANCOVA – Clothing Related Difficulties in Emotion Regulation Scale (CRDERS) POSTTEST.

Cases	Sum of Squares	df	Mean Square	*F*	*P*	*η* ^2^_*p*_
Group	94.185	1	94.185	335.157	<.001	0.756
Gender	2.453	1	2.453	8.728	.004	0.075
CRDERSPRETEST	0.329	1	0.329	1.172	.281	0.011
Group × gender	2.351	1	2.351	8.367	.005	0.072
Residuals	30.350	108	0.281			

Type III sum of squares.

In summary, despite the initial statement claiming no significant difference, the data clearly indicate that CBT significantly enhanced participants’ emotional regulation abilities related to impulsive clothing buying. Participants who received CBT showed markedly reduced emotional dysregulation compared to those in the control group, underscoring the therapy’s effectiveness in helping individuals manage the emotional triggers that often precede impulsive purchasing behavior.

A total of 9 participants (8 from the CBT group and 1 from the control group) withdrew before completing the post-intervention assessment. Missing data were handled using the intention-to-treat principle, ensuring that all participants initially randomized were included in the analysis. Where feasible, missing posttest values were estimated using the last observation carried forward approach. Sensitivity checks were also conducted using per-protocol analyses to confirm that the exclusion of incomplete cases did not substantially alter the statistical outcomes. Attrition analyses revealed no significant baseline differences between completers and non-completers, suggesting that data were missing at random and unlikely to bias the final results.

## 7. Discussion

This research provides compelling evidence supporting the efficacy of CBT in mitigating impulsive clothing purchases among adolescents. Participants who received CBT demonstrated a marked reduction in impulsive buying tendencies compared to those in the control group, indicating that CBT is an effective intervention for modifying maladaptive consumer behaviors. This finding is consistent with the theoretical framework of CBT, which emphasizes restructuring dysfunctional thought patterns and reinforcing adaptive behaviors through cognitive and behavioral skill-building strategies.^[34]^

A key mechanism underlying this improvement was the significant reduction in cognitive distortions related to impulsive clothing purchases. The posttest results revealed that participants exposed to CBT reported substantially lower levels of irrational beliefs and distorted thinking patterns than their counterparts in the control group. This outcome reinforces the proposition that cognitive distortions serve as cognitive antecedents to impulsive consumer actions, and that targeting these maladaptive cognitions is central to behavioral change.^[35,36]^ Previous studies have similarly reported that CBT and its derivatives, such as rational emotive behavior therapy, effectively reduce irrational beliefs and cognitive distortions across diverse populations and behavioral contexts.^[37,38]^ The present findings extend this empirical support to the specific domain of impulsive clothing purchases among adolescents, demonstrating CBT’s capacity to address the cognitive underpinnings of compulsive buying behaviors.

Furthermore, the findings revealed a significant enhancement in emotional regulation skills among participants who underwent CBT. The experimental group exhibited strong emotional control and stability, as evidenced by minimal variability in posttest scores, compared to the control group, which continued to experience considerable emotional dysregulation. This result underscores the effectiveness of CBT in fostering adaptive emotional regulation a critical protective factor against impulsive behavior. Consistent with prior studies, CBT appears to equip individuals with tools to identify, understand, and reframe maladaptive emotional responses, thereby reducing the likelihood of emotionally driven purchases.^[39–41]^ Improved emotional regulation, in turn, likely contributed to the observed decline in impulsive buying, as emotional triggers are often central to spontaneous consumer decisions.

Interestingly, pretest scores on impulsivity, cognitive distortions, and emotion regulation did not significantly influence post-intervention outcomes, suggesting that the improvements observed were attributable primarily to the CBT intervention rather than to baseline differences or natural fluctuations. This finding enhances the internal validity of the study and supports the inference that CBT was the principal causal factor behind the observed behavioral and emotional improvements.^[42–44]^

In conclusion, this research strengthens the growing body of literature validating CBT as a robust intervention for curbing impulsive clothing purchases among adolescents. By simultaneously addressing cognitive distortions and emotional dysregulation, CBT offers a comprehensive framework for promoting rational decision-making and emotional balance in consumer contexts. These findings highlight the importance of integrating CBT-based interventions into school and community programs designed to enhance self-control, financial responsibility, and mental wellbeing among young people. Future research should examine the long-term sustainability of these effects and explore how CBT may be adapted to address other forms of impulsive or compulsive behaviors across diverse adolescent populations.

## 8. Conclusion

This research demonstrates that CBT is a powerful and effective intervention for mitigating impulsive clothing purchases and the cognitive distortions that sustain such behavior, while simultaneously enhancing emotional regulation among adolescents. The intervention’s positive effects across behavioral, cognitive, and emotional domains underscore the comprehensive benefits of CBT in addressing problematic consumer habits and the underlying psychological mechanisms that drive them. These findings advocate for the integration of CBT-based strategies into adolescent development programs, particularly those aimed at promoting mental wellbeing, self-regulation, and informed decision-making. By equipping young individuals with cognitive and emotional tools for managing impulses, CBT supports the cultivation of adaptive behaviors essential for navigating the consumer and social pressures characteristic of adolescence.

### 8.1. Implications of the study

The findings from this study carry important implications for adolescent mental health interventions, behavioral research, and psychoeducational practice. By empirically demonstrating the effectiveness of CBT in reducing impulsive clothing purchases, restructuring cognitive distortions, and improving emotional regulation, the study reinforces CBT as a comprehensive and adaptable framework for addressing adolescent behavioral challenges. These results suggest that CBT’s utility extends beyond traditional clinical contexts to encompass everyday consumer behaviors that are both emotionally charged and cognitively reinforced such as impulsive or compulsive spending. In this way, the study advances understanding of the cognitive-emotional mechanisms underlying maladaptive consumer patterns in youth populations.

Moreover, the results highlight the importance of equipping adolescents with cognitive and emotional self-regulation strategies during a critical stage of identity development and social adaptation. Impulsive clothing purchases, often driven by distorted self-perceptions and poor emotional regulation, can adversely affect psychological wellbeing, self-esteem, and financial responsibility. Implementing structured CBT-based interventions within school, clinical, and community settings can serve both preventive and corrective functions, empowering adolescents to develop healthier coping and decision-making skills. The demonstrated success of CBT in transforming thought patterns and emotional responses positions it as a dual-action model ideally suited for inclusion in adolescent-focused behavioral health frameworks and educational curricula.

### 8.2. Recommendations

Based on the findings of this study, several recommendations are proposed for practitioners, educators, and researchers concerned with adolescent wellbeing and behavioral regulation.

#### 8.2.1. Integration of CBT into school-based programs

Educational institutions should consider incorporating CBT principles into life skills, guidance, and counseling curricula. Structured CBT modules can help adolescents identify and modify impulsive thought patterns and emotional triggers that lead to unplanned or excessive spending. This integration would also strengthen students’ broader self-management and problem-solving abilities, fostering responsible consumer and emotional habits.

#### 8.2.2. Training and capacity building for counselors and teachers

Professional development initiatives should be established to train school counselors, psychologists, and educators in the application of CBT techniques tailored to adolescent populations. Such training would enable these professionals to deliver early interventions that address not only academic or social difficulties but also maladaptive consumer and emotional behaviors.

#### 8.2.3. Development of community-based CBT interventions

Community organizations and youth development agencies should collaborate to design accessible CBT-informed workshops and mentorship programs. These initiatives could target at-risk youth populations and provide practical guidance on emotion regulation, financial decision-making, and self-control within real-life consumer contexts.

#### 8.2.4. Policy inclusion and funding support

Educational and mental health policymakers should recognize the relevance of CBT-based interventions in promoting youth wellbeing and financial responsibility. Adequate funding and institutional support should be allocated to implement such programs sustainably across schools and community centers, particularly in regions where impulsive consumer behavior among adolescents is prevalent.

#### 8.2.5. Directions for future research

Subsequent studies should explore the long-term impact of CBT on impulsive consumer behaviors and investigate potential moderating factors such as socioeconomic status, peer influence, and digital media exposure. Longitudinal and cross-cultural studies could further validate the generalizability of these findings and enhance the theoretical understanding of the interplay between cognitive distortions, emotion regulation, and impulsive decision-making among adolescents.

### 8.3. Limitations of the study

Although this study provides valuable insights into the effectiveness of CBT in reducing impulsive clothing purchases, cognitive distortions, and emotional dysregulation among adolescents, certain limitations should be acknowledged.

First, the study relied primarily on self-report measures, which may be influenced by social desirability bias or participants’ subjective perceptions of their behaviors and emotional states. While standardized and validated instruments were used, the inclusion of observational or behavioral data in future research would strengthen the objectivity and reliability of outcomes.

Second, the study’s sample was drawn from a specific adolescent population within a particular cultural and educational context. Consequently, the findings may not be entirely generalizable to adolescents from different sociocultural or economic backgrounds. Future investigations should therefore include more diverse and representative samples across multiple settings to enhance external validity.

Third, the posttest-only analysis design limited the assessment of the long-term sustainability of CBT’s effects. Longitudinal follow-up studies are recommended to evaluate whether the improvements in impulsive buying behavior, cognitive distortions, and emotion regulation persist beyond the immediate post-intervention period.

Fourth, although gender differences and interactions were examined, other potentially influential variables, such as socioeconomic status, parental influence, peer pressure, or exposure to digital marketing, were not controlled for. Including these variables in future studies could provide a more comprehensive understanding of the psychosocial and contextual factors influencing impulsive consumer behavior.

Fifth, the controlled experimental setting enhanced internal validity but may not fully capture real-world consumer conditions where impulsive buying behaviors naturally occur. Employing ecologically valid research designs, such as simulated online shopping environments or field-based behavioral tracking, would improve the general applicability of results.

Finally, trial registration was not conducted prospectively. This occurred because the study began as part of a broader educational intervention initiative before formal classification as a clinical or behavioral trial was considered. Consequently, registration was completed retrospectively after the intervention phase had commenced. While all ethical guidelines were observed and institutional approval was obtained prior to data collection, future studies should ensure prospective trial registration in line with international research transparency standards.

## Author contributions

**Conceptualization:** Ekwutosi Beatrice Oluah, Uzoamaka C. Ogor, Daniel Okon, Lilian C. Ozoemena.

**Data curation:** Ekwutosi Beatrice Oluah, Daniel Okon, Damian C. Ncheke, Lilian C. Ozoemena, D. C. Ezeuhara, Daniel Omutal.

**Formal analysis:** Ekwutosi Beatrice Oluah, Uzoamaka C. Ogor, Daniel Okon, Damian C. Ncheke, Lilian C. Ozoemena, Uche Hope Ekwueme, D. C. Ezeuhara, Oluchi P. Ofozoba, Chinonye Lilian Opara, Daniel Omutal.

**Funding acquisition:** Ekwutosi Beatrice Oluah, Uzoamaka C. Ogor, Daniel Okon, Uche Hope Ekwueme, Nkechi T. Egenti, D. C. Ezeuhara, Chinonye Lilian Opara, Daniel Omutal.

**Investigation:** Ekwutosi Beatrice Oluah, Uzoamaka C. Ogor, Elizabeth Nkechi Ugwu, Lilian C. Ozoemena, Uche Hope Ekwueme, Nkechi T. Egenti, Chinonye Lilian Opara.

**Methodology:** Uzoamaka C. Ogor, Daniel Okon, Damian C. Ncheke, Uche Hope Ekwueme, D. C. Ezeuhara, Daniel Omutal.

**Project administration:** Ekwutosi Beatrice Oluah, Margaret Ajanigo Atadoga, Damian C. Ncheke, Nkechi T. Egenti, Patience Nwobodo.

**Resources:** Uzoamaka C. Ogor, Daniel Okon, Ngozi Ngwanna Awoke.

**Software:** Elizabeth Nkechi Ugwu, Margaret Ajanigo Atadoga, Daniel Okon, Ngozi Ngwanna Awoke, Nkechi T. Egenti, Oluchi P. Ofozoba, Patience Nwobodo.

**Supervision:** Uzoamaka C. Ogor, Elizabeth Nkechi Ugwu, Margaret Ajanigo Atadoga, Ngozi Ngwanna Awoke, Nkechi T. Egenti, Chinonye Lilian Opara.

**Validation:** Elizabeth Nkechi Ugwu, Uche Hope Ekwueme, Nkechi T. Egenti, Oluchi P. Ofozoba, Chinonye Lilian Opara.

**Visualization:** Elizabeth Nkechi Ugwu, Margaret Ajanigo Atadoga, Ngozi Ngwanna Awoke, Oluchi P. Ofozoba, Patience Nwobodo.

**Writing – original draft:** Elizabeth Nkechi Ugwu, Margaret Ajanigo Atadoga, Ngozi Ngwanna Awoke, Patience Nwobodo.

**Writing – review & editing:** Elizabeth Nkechi Ugwu, Margaret Ajanigo Atadoga, Daniel Omutal, Patience Nwobodo.
